# Innocent until proven guilty: acute on chronic tubal torsion mimicking pelvic inflammatory disease

**DOI:** 10.1093/jscr/rjab187

**Published:** 2021-05-17

**Authors:** Scott Taylor, Hong Lee, Rajeev Singh

**Affiliations:** Department of Obstetrics and Gynaecology, Joondalup Health Campus, Joondalup, WA, Australia; Department of Obstetrics and Gynaecology, Joondalup Health Campus, Joondalup, WA, Australia; Department of Obstetrics and Gynaecology, Joondalup Health Campus, Joondalup, WA, Australia

**Keywords:** isolated tubal torsion, para-tubal cysts, hydrosalpinx

## Abstract

Acute pelvic pain is a common complaint in reproductive age women and has a large differential diagnosis. Decision for conservative vs. surgical management is often dependent on clinical, biochemical and imaging findings. Isolated tubal torsion is a rare cause of pelvic pain that requires prompt diagnosis and surgical management to avoid morbidity. Here, we report non-pregnant women of reproductive age presenting with acute lower abdominal pain. Raised inflammatory markers and ultrasound findings prompted management for Pelvic Inflammatory Disease. Despite some improvement with antibiotics, the patient had ongoing symptoms. At surgery, bilateral para-tubal cysts and a left sided hydrosalpinx were found, along with an isolated left tubal torsion. Isolated tubal torsion most commonly occurs in reproductive aged women, and risk factors include intrinsic tubal pathology and extrinsic lesions. Clinically, biochemically and radiographically, it is often indistinguishable from other pelvic pathology, potentially leading to diagnostic delay, and necrosis of the tube.

## INTRODUCTION

Acute pelvic pain in women of reproductive age is a common clinical presentation. The differential diagnosis in a non-pregnant woman of reproductive age is broad and includes ovarian torsion, cyst accident (rupture of follicle or cyst), pelvic inflammatory disease (PID), appendicitis, intestinal obstruction or perforation, renal stones or cystitis [1].

Decision for conservative management vs. diagnostic surgery in a woman with suspected pelvic pathology is dependent on a combination of factors, including the patient’s age, severity of pain, associated symptomology, risk factors, inflammation and tumour markers and imaging findings. In many cases, diagnostic laparoscopy is the only definitive method for confirming a suspected diagnosis.

Isolated fallopian tubal torsion, characterized by tubal rotation around its own axis without ipsilateral ovarian torsion, is a rare but important cause of acute pelvic pain in this population, with the potential for significant morbidity if surgical diagnosis and correction is delayed [1].

## CASE REPORT

A 44-year-old female presented to the Emergency Department with lower abdominal pain and ‘feeling of pressure in rectum’ during bowel motions associated with an increased urge to defecate. She had not been sexually active for more than 10 years. On examination, she was tender in the lower abdomen bilaterally, and cervical motion tenderness was elicited. Bloodwork demonstrated a raised white cell count and C-reactive protein and negative beta hcg. She was diagnosed with PID and commenced on intravenous antibiotics.

**Figure 1:**
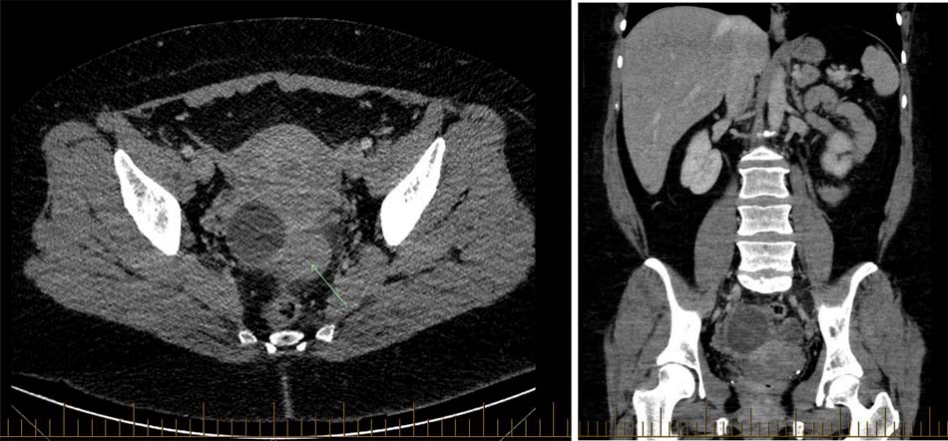
CT abdomen demonstrating pelvic pathology.

**Figure 2:**
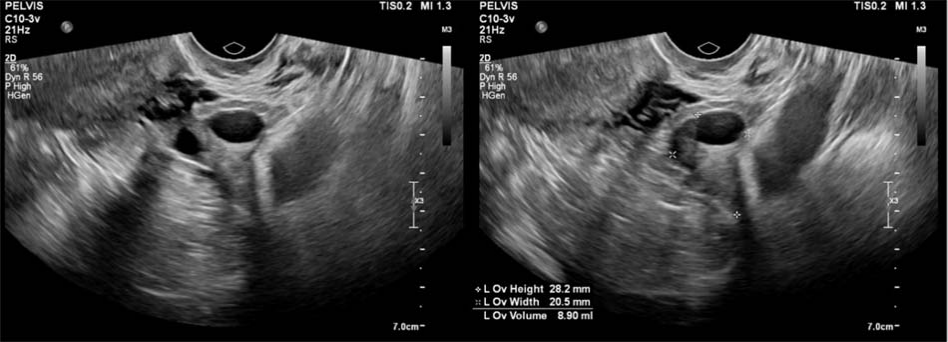
Ultrasound imaging of the right ovary, demonstrating a simple cyst.

**Figure 3:**
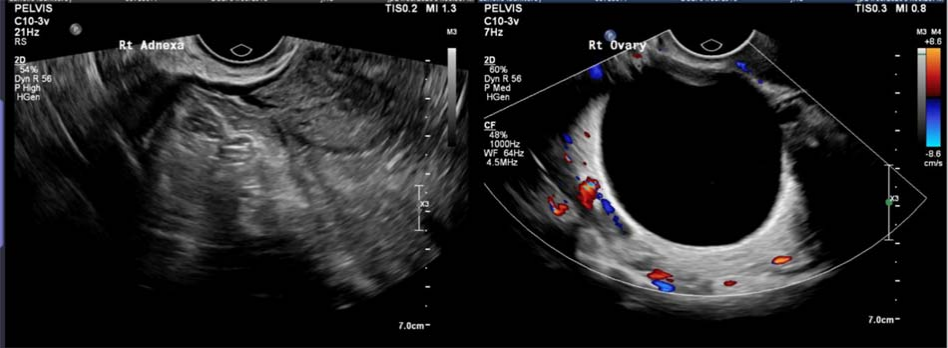
Left ovary with suspected tubo-ovarian complex.

CT abdomen showed two cystic structures in the pelvis, and follow-up ultrasound demonstrated a 5.8-cm unilocular simple cyst contained within the right ovary, and a 3-cm left tubo-ovarian complex with associated hydrosalpinx (Figs 1–3).

Ovarian tumour markers, blood cultures, vaginal swabs and STI screening were negative. The patient clinically improved and on Day 3 was discharged on oral antibiotics (metronidazole and doxycycline as per local protocol for PID treatment) with outpatient follow-up arranged. Subsequent ultrasound 3 months later suggested a 5-cm simple right ovarian cyst and a left hydrosalpinx, and she had ongoing intermittent pain. She was taken to theatre for a bilateral salpingectomy and right cystectomy.

**Figure 4:**
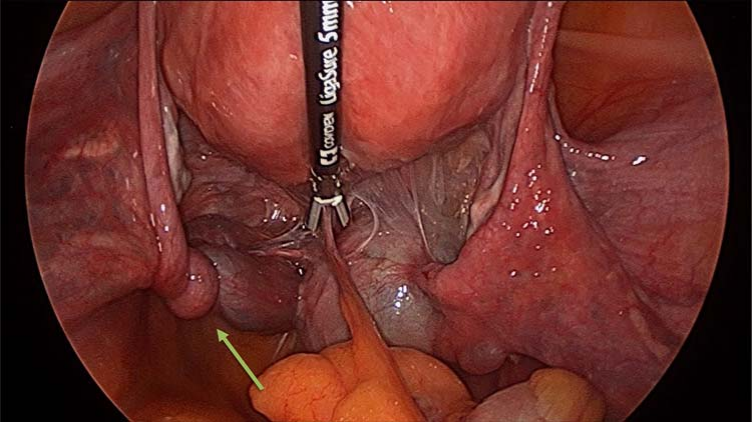
Left tubal torsion.

**Figure 5:**
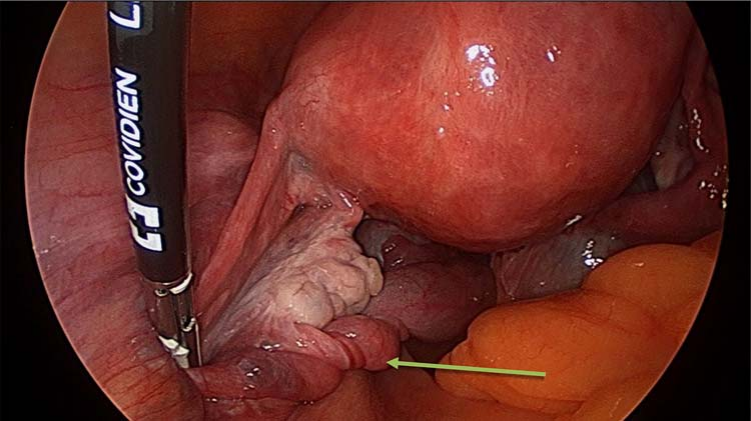
The left tube was torted three times.

**Figure 6:**
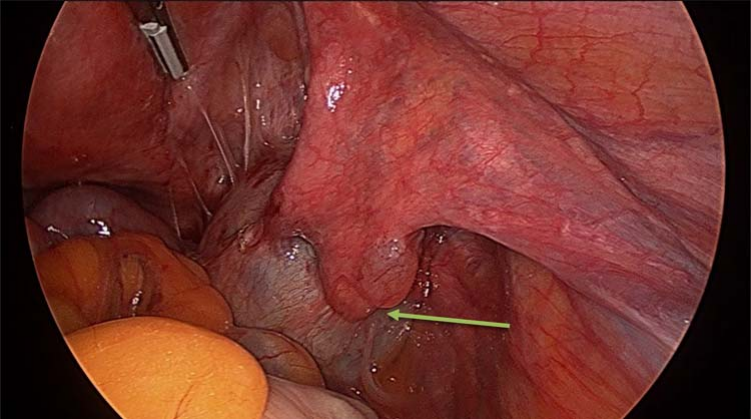
Right para-tubal cyst, implanted to the pouch of Douglas and right ovarian fossa.

Operation findings consisted of left hydrosalpinx and para-tubal cyst adhered to pouch of douglas, with the left tube torted 3 times (Figs 4 and 5). There was a 5-cm para-tubal cyst on the right tube adhered to right side of pelvis/rectum/pouch of douglas (Fig. 6). The ovaries appeared normal.

## DISCUSSION

Isolated tubal torsion is an uncommon, but important cause of acute pelvic pain in women, with a reported prevalence of 1 in 1.5 million [1]. The presentation is non-specific; patients often present with sudden onset pain, which can be sharp or dull, radiating to the thigh or groin [1]. Associated signs and symptoms may include nausea and vomiting, localized peritinism or palpable adnexal mass [1].

The aetiology of tubal torsion is poorly understood, however, predisposing factors include hydrosalpinx, para tubal cysts, ectopic pregnancy, ovarian mass or extrinsic lesions (adhesions, endometriosis) [1, 2]. Other risk factors that have been proposed include pregnancy, trauma or sudden body movements [2]. Tubal torsion most commonly affects the right side, possibly because of partial immobilization of the left tube secondary to proximity of the sigmoid mesentery [1].

In this case however, the torsion was left sided. From an aetiological perspective, there were two risk factors for isolated tubal torsion, the hydrosalpinx and the small para-tubal cyst.

Para tubal cysts arise from the broad ligament between the ovary and tube, and most commonly originate from the remnants of the Mullerian or Wolffian ducts. They account for 10–20% of adnexal masses and are most commonly found in the third and fourth decades of life [3]. They are usually asymptomatic, but possible complications include cyst enlargement (79.62%), adnexal torsion (18.51%), haemorrhage (7.4%) and rupture (1.85%) [1]. They are rarely malignant, though borderline tumours have been reported [4].

A hydrosalpinx is a distally obstructed fallopian tube filled with serous or clear fluid. While it can occur in children and adolescents associated with tubal agenesis, it most commonly occurs in reproductive aged women, seen as a complication of infection (including PID), surgery or ovarian cysts [5, 6]. PID is an infection of the female upper reproductive tract which is commonly associated with chlamydia and gonorrhoea STIs, but can also occur in non-sexually active women [7].

Trans-vaginal ultrasound is the gold standard modality for characterizing an adnexal mass, though para-tubal cysts and ovarian cysts can be difficult to distinguish [2, 4]. Unfortunately, the diagnosis of tubal torsion is difficult to make with imaging [2]. Arterial and venous flow to an adnexal mass may be demonstrated with colour Doppler, through the presence of flow does not exclude torsion [2]. The presence of sonographic features in conjunction with the patient’s symptoms should guide the need for diagnostic surgery.

Diagnostic laparoscopy is the only method of confirming tubal torsion [6]. Surgical technique for correcting isolated tubal torsion may include conservative (detorsion) or definitive management (salpingectomy). Choice of management will be influenced by patient factors including age, completion of fertility and symptoms, as well as surgical factors including ease of detorsion, appearance of revascularization and associated pelvic pathology. Because isolated tubal torsion is difficult to distinguish clinically from differential diagnoses, delay in surgical exploration often leads to necrotic appearances of the tube, requiring salpingectomy [5].

As isolated tubal torsion is rare and the presentation is non-specific, it is commonly misdiagnosed clinically, as was the case here. When she presented to emergency, this woman was given a presumptive diagnosis of PID and managed accordingly. Presumptive diagnoses of PID in the clinical setting leads to early antibiotic administration which can help to prevent PID complications, but the diagnosis carries negative connotations which can lead to psychological and relationship stress. Diagnosis is usually confirmed by positive cervical swabs for chlamydia/gonorrhoea or positive vaginal swabs, though it can occur in the absence of an STI [7]. Imaging demonstrating tubo-ovarian abscess, a severe complication of PID, compliments the diagnosis. Despite no risk factors for PID and negative endocervical and vaginal swabs, the combination of raised inflammatory makers and ultrasound findings were enough that the diagnosis of PID was retained for this woman until ultimately being refuted at time of laparoscopy.
